# An *in situ* phase transformation induced mesoporous heterointerface for the alkaline hydrogen evolution reaction†

**DOI:** 10.1039/d4ra02063d

**Published:** 2024-05-07

**Authors:** Zhiping Lin, Zongpeng Wang, Tianchen Jin, Ting Jiang, Longfei Ding, Shijie Shen, Jitang Zhang, Wenwu Zhong

**Affiliations:** a School of Material Science and Engineering, Taizhou University No. 1139, Shifu Road Taizhou 318000 China zhangjt@tzc.edu.cn zhongww@tzc.edu.cn; b School of Chemical Engineering and Technology, China University of Mining and Technology No. 1, Daxue Road Xuzhou 221116 China; c School of Material Science and Engineering, Central South University No. 932, Lushan South Road Changsha 410083 China; d ERA Co, Ltd No. 1118, Huangjiao Road Taizhou 318020 China; e College of Chemical and Biological Engineering, Zhejiang University No. 866, Yuhangtang Road Hangzhou 310058 China

## Abstract

The phase structure of a catalyst plays a crucial role in determining the catalytic activity. In this study, a facile phosphorization process is employed to achieve the *in situ* phase transformation from single-phase Co_3_O_4_ to CoO/CoP hybrid phases. Characterization techniques, including XRD, BET, SEM, and TEM, confirm the retention of the mesoporous nature during the phase transformation, forming porous CoO/CoP heterointerfaces. Strong charge transfer is observed across the CoO/CoP heterointerface, indicating a robust interaction between the hybrid phases. The CoO/CoP hybrid exhibits significantly enhanced catalytic activity for the alkaline hydrogen evolution reaction (HER) compared to pristine Co_3_O_4_. Density Functional Theory (DFT) calculations reveal that the elimination of the band gap in the spin-down band of Co in CoO/CoP contributes to the observed high HER activity. The findings highlight the potential of CoO/CoP hybrids as efficient catalysts for HER, and contribute to the advancement of catalyst design for sustainable energy applications.

## Abstract 2

*In situ* phase transformation is achieved from single-phase Co_3_O_4_ to CoO/CoP hybrid phases, which acts as a highly efficient electrocatalyst for the hydrogen evolution reaction.

## Graphical abstract

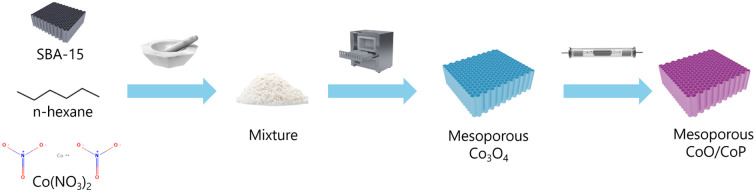

## 1. Introduction

Hydrogen is considered a clean and versatile energy carrier,^1–5^ and its production through water electrolysis, driven by renewable energy sources, offers a promising solution to reduce our reliance on fossil fuels and mitigate environmental impact.^6–14^ The hydrogen evolution reaction (HER) is a crucial process in the field of hydrogen production, which takes place at the cathode, where protons form hydrogen gas. HER can occur in both acidic and alkaline environments. Alkaline conditions are considered more environmentally friendly due to the avoidance of corrosive acidic electrolytes.^15–22^ Furthermore, the other half reaction of water splitting, the oxygen evolution reaction (OER) exhibits more favorable kinetics in alkali than in acid.^23–29^ Thus, alkaline HER can enable efficient, cost-effective, and environmentally friendly hydrogen production. Efficient and cost-effective catalysts are crucial for accelerating the HER and improving the overall efficiency of hydrogen production. Noble metals such as platinum are effective catalysts but are expensive.^30–34^ Considerable efforts have been dedicated to working on developing alternative, non-precious metal catalysts, including various metal oxides,^35–37^ sulfides,^38–40^ phosphides^41–43^ and so on.

Cobalt oxides have recently garnered attention for their potential applications in water splitting.^44–48^ Several factors contribute to their attractiveness for large-scale use: firstly, cobalt is abundant on Earth, enhancing the feasibility of widespread applications. Secondly, the variable oxidation states of cobalt in cobalt oxides allow for the acceptance and donation of electrons during the reaction, resulting in a unique electronic structure that can be finely tuned. Strategies such as the incorporation of nitrogen or phosphorus have been explored to enhance overall catalytic activity. Thirdly, cobalt oxides are usually effective involving the participation of hydroxide ions, which is essential in alkaline HER. However, despite their catalytic properties being extensively studied for OER, the catalytic activity of cobalt oxides for the HER remains relatively low and less explored. Considering the mentioned advantages, the development of highly efficient HER catalysts based on cobalt oxides is of paramount importance. Nevertheless, this pursuit faces challenges in achieving the high efficiency of cobalt-oxides-based catalysts, understanding the underlying reaction mechanisms, and ensuring sustained long-term performance. Addressing these challenges will be crucial for advancing the practical applications of cobalt oxides in the field of water splitting. Meanwhile, cobalt phosphide (CoP) has also emerged as a promising catalyst, due to its favorable catalytic activity, abundance, and versatility of modulation. CoP samples with various morphologies such as nanorod,^49^ nanoflower,^50^ nanotube^51^ and others have been demonstrated with excellent HER activities. CoP has also been doped with N,^52^ Cu,^53^ W^54^ and other elements to achieve better performance. Besides those strategies, forming heterointerface has been recognized as a promising method to boost the catalytic activity by introducing interaction between the two phases side by side.^55–58^ Normally, strong charge transfer and build-in field can be observed across the heterointerface, which will regulate the electron properties and optimize the adsorptive features of the interface, thus realizing advanced catalytic activity. However, the construction of heterointerfaces by sequentially overlaying one phase onto another often results in insufficient contact and mechanical stability. The conversion of one individual phase into hybrid phases simultaneously to fabricate *in situ* heterointerfaces remains a challenge.

In this work, we have successfully utilized a facile phosphorization process to achieve the *in situ* transformation of a single-phase Co_3_O_4_ material into CoO/CoP hybrid phases. Notably, the mesoporous nature of Co_3_O_4_ is preserved throughout the phase transformation, leading to the formation of porous CoO/CoP heterointerfaces. The observed strong charge transfer across these heterointerfaces indicates a robust interaction between the hybrid phases. Importantly, CoO/CoP exhibits significantly enhanced catalytic activity for the hydrogen evolution reaction (HER) compared to pristine Co_3_O_4_. DFT calculations provide valuable insights into the observed high HER activity, revealing that the elimination of the band gap in the spin-down band of Co in CoO/CoP plays a crucial role in the improved catalytic performance. This work highlights the potential of CoO/CoP hybrids as efficient catalysts for HER.

## 2. Experiments

### 2.1 Synthesis of Co_3_O_4_

Mesoporous Co_3_O_4_ was synthesized utilizing the two-solvent method,^59^ employing SBA-15 as template. The typical fabrication procedure is outlined as follows: 40 mL of *n*-hexane was introduced into a beaker, followed by the addition of 0.8 g of purchased SBA-15. After thorough stirring at room temperature, a measured quantity of Co(NO_3_)_2_ solution was incrementally added. The resulting mixture was stirred continuously for 20 hours, followed by ambient temperature washing and drying. Subsequently, the obtained powder was heated in a muffle furnace to 300 °C, maintaining this temperature for 5 hours. Multiple etching steps with NaOH solution, were employed to remove the silica template. After thorough cleaning and drying, mesoporous Co_3_O_4_ was successfully obtained.

### 2.2 Synthesis of CoO/CoP

The CoO/CoP was prepared following a phosphorization procedure as shown in Fig. S1.† In an exemplary process, 30 mg mesoporous Co_3_O_4_ were placed into a quartz boat within a tubular furnace, while another quartz boat containing 600 mg NaH_2_PO_2_·H_2_O was positioned in the same furnace. In the tube, Co_3_O_4_ was put at the downstream side, while NaH_2_PO_2_·H_2_O at the upstream side. The tubular furnace was gradually heated to 350 °C at a rate of 5 °C min^−1^, and after maintaining this temperature for one hour, it was slowly cooled to room temperature. This controlled thermal treatment resulted in the formation of CoO/CoP.

### 2.3 Characterization methods

X-ray diffraction (XRD) analysis was undertaken utilizing a Bruker-D8 Advance instrument equipped with Cu Kα radiation. Scanning electron microscopy (SEM) images were acquired using a Hitachi S-4800 apparatus. Transmission electron microscopy (TEM), scanning transmission electron microscopy (STEM), and selected area electron diffraction (SAED) measurements were carried out employing the FEI Tecnai G2 F20. Energy-dispersive X-ray spectroscopy (EDS) analysis was conducted using the Super-X EDS system. X-ray photoelectron spectroscopy (XPS) spectra were obtained utilizing the Thermo Scientific K-Alpha, with an Al Kα radiation source (*hν* = 1486.6 eV). Brunauer–Emmett–Teller (BET) measurements were performed on the Micromeritics ASAP 2460 3.01.

### 2.4 Electrochemical measurements

The electrochemical assessments were executed employing a CHI 660E electrochemical workstation, employing a standard three-electrode configuration at room temperature. Specifically, a glass carbon electrode served as the substrate for the catalysts and functioned as the working electrode. A graphite rod was utilized as the counter electrode, while the Ag/AgCl electrode was employed as the reference electrode in a 1 M KOH electrolyte. To fabricate the working electrode, a composite mixture of 5 mg catalyst, 750 μL alcohol, 250 μL isopropanol, and 50 μL Nafion solution (5 wt%) was thoroughly homogenized through ultrasonic treatment for a duration of half an hour. Subsequently, 5 μL of the thus-prepared suspension was deposited onto the meticulously cleaned surface of the glass carbon electrode. Following natural drying, the resulting working electrode was rendered suitable for implementation in subsequent electrochemical tests.

The hydrogen evolution reaction (HER) efficacy of the respective catalysts was assessed in 1 M KOH solutions. Linear sweep voltammetry (LSV) curves were recorded at a sweep rate of 5 mV s^−1^ with *iR* correction. Tafel slopes were determined from the acquired LSV curves. Cyclic voltammetry (CV) measurements spanned the range of 0.11–0.21 V relative to the reversible hydrogen electrode, with the sweep speed incrementally adjusted from 20 to 200 mV s^−1^. Electrochemical impedance spectroscopy was conducted under a constant overpotential of 120 mV, covering a frequency range from 100 kHz to 0.1 Hz.

## 3. Results and discussion

**Fig. 1:**
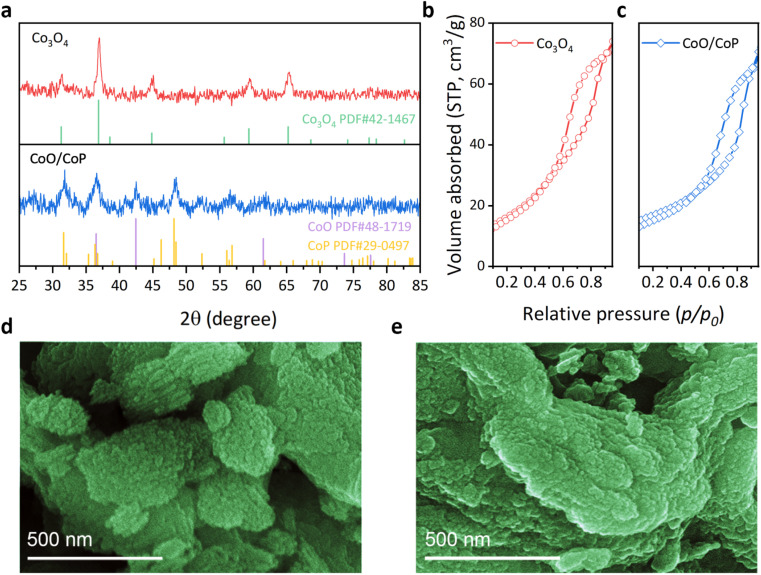
(a) XRD patterns of Co_3_O_4_ (red) and CoO/CoP (blue), respectively. (b and c) BET measurements of Co_3_O_4_ (red) and CoO/CoP (blue), respectively. (d and e) SEM photos of Co_3_O_4_ and CoO/CoP, respectively.

The crystal structure of the samples was investigated through X-ray diffraction (XRD), as depicted in Fig. 1a. The Co_3_O_4_ sample exhibits diffraction peaks at 31.3, 36.9, 38.5, 44.8, 55.7, 59.4, and 65.2°, aligning with the standard PDF card of Co_3_O_4_ (No. 42-1467). No other diffraction peaks are observable, attesting to the high phase purity of the as-prepared Co_3_O_4_. Specifically, the identified diffraction peaks correspond to the (220), (311), (222), (400), (422), (511), and (440) crystal facets of Co_3_O_4_, respectively. In the case of the CoO/CoP sample, two distinct sets of diffraction peaks were discerned. The set at 36.5, 42.4, and 61.5° aligns with the standard PDF card of CoO (No. 48-1719), corresponding to the (111), (200), and (220) crystal facets, respectively. Conversely, the second set at 31.6, 32, 38.9, 46.2, 48.1, 48.4, 52.3, 56.4, and 56.8° matches with the standard PDF card of CoP (No. 29-0497), corresponding to the (011), (002), (200), (112), (211), (202), (103), (020), and (301) facets of CoP, respectively. Notably, the absence of prominent Co_3_O_4_ peaks indicates the thoroughness of the phosphorization process. The XRD results suggest an *in situ* transformation of Co_3_O_4_ into CoO/CoP compounds following phosphorization.

The porous nature of the as-prepared Co_3_O_4_ samples were revealed through the Brunauer–Emmett–Teller (BET) method, as depicted in Fig. 1b and c. Co_3_O_4_ exhibits a specific surface area of 59 m^2^ g^−1^, while 42.2 m^2^ g^−1^ for CoO/CoP, suggesting only some portion of porous channels are destructed after phosphorization. The morphology of the synthesized samples was scrutinized through scanning electron microscopy (SEM) images, as depicted in Fig. 1d, e and S2.† The pristine Co_3_O_4_ manifests a morphology comprised of stacked flakes, with an outline size ranging from 1 to 4 μm. A congruent morphology has been previously documented in studies utilizing the SBA-15 as templates.^60,61^ Upon phosphorization, CoO/CoP retains a similar morphology, albeit with more dispersed exfoliated small flakes and softer edges. These observations suggest the preservation of the structural framework during the *in situ* phase transformation. It is noteworthy that the SEM images do not reveal mesoporous channels, attributable to the diminutive pore size of ∼7 nm.

**Fig. 2:**
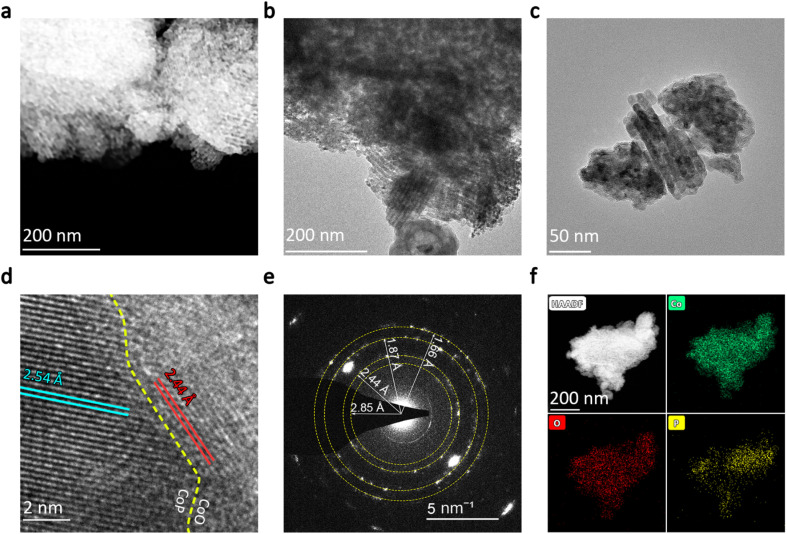
(a) STEM photo of CoO/CoP. (b and c) TEM photos of CoO/CoP at different magnifications. (d) HRTEM photo of CoO/CoP. (e) SAED pattern of CoO/CoP. (f) Element mapping of CoO/CoP.

To further explore the mesoporous channels and the crystal structure of the synthesized CoO/CoP, transmission electron microscopy (TEM) characterizations were conducted. The scanning transmission electron microscopy (STEM) image of CoO/CoP is presented in Fig. 2a, while TEM images at various magnifications are displayed in Fig. 2b and c. Both STEM and TEM images distinctly exhibit mesoporous channel structures, originated from the removal of the SBA-15 template. Analogous mesoporous structures have been documented in prior studies.^62–65^ To investigate the phase transformation resulted from phosphorization, high-resolution TEM images were obtained. As depicted in Fig. 2d, a well-defined CoO/CoP heterointerface is formed in CoO/CoP. The identified lattice spacings of 2.54 and 2.44 Å (indicated by blue and red lines, respectively) correspond to the (200) and (111) crystal planes of CoP and CoO, respectively. To provide additional evidence for the existence of the CoO/CoP heterointerface, selected area electron diffraction (SAED) was performed. The SAED rings in Fig. 2e reveal the coexistence and polycrystalline nature of CoO/CoP. The specified distances of 2.85, 1.87, and 1.66 Å align with the spacings of (011), (211), and (020) crystal facets in CoP, while the 2.44 Å distance matches the (111) facet spacing in CoO. Energy-dispersive X-ray spectroscopy (EDS) element mapping was also employed to investigate the element distribution in CoO/CoP, as shown in Fig. 2f. It is evident that the Co and O elements are homogeneously distributed throughout the sample. However, the distribution of the P element exhibits noticeable segregation, serving as additional compelling evidence for the CoO/CoP heterointerface. One may speculate that O should distribute separately from P, by simply examining the chemical denotation of “CoO/CoP”. The absence of O segregation in the EDS mapping is attributed to the well-known adsorption tendency of O species on CoP, which is evidenced below. These analyses, in conjunction with the XRD, BET, SEM, and TEM results, unequivocally affirm the transformation of mesoporous Co_3_O_4_ into mesoporous CoO/CoP heterointerface material following phosphorization.

**Fig. 3:**
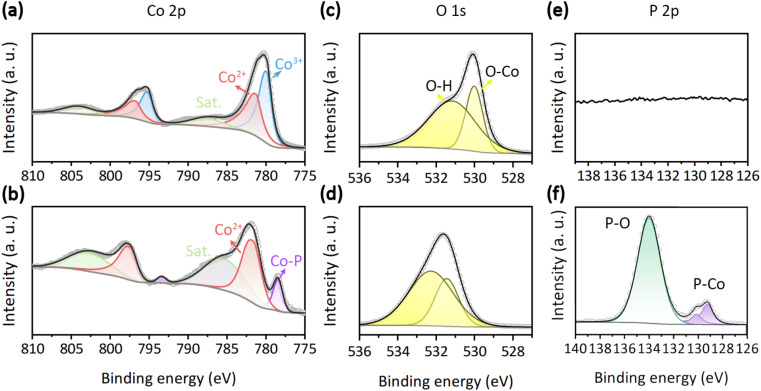
(a and b) Co 2p XPS spectra of Co_3_O_4_ and CoO/CoP, respectively. (c and d) O 1s XPS spectra of Co_3_O_4_ and CoO/CoP, respectively. (e and f) P 2p XPS spectra of Co_3_O_4_ and CoO/CoP, respectively.

The chemical state is a crucial parameter in determining the catalytic activity of an electrocatalyst. The full X-ray photoelectron spectroscopy (XPS) survey of the synthesized samples is presented in Fig. S4.† All XPS spectra are aligned using the C 1s peak at 284.8 eV (Fig. S3†). For Co_3_O_4_, discernible peaks are observed only for Co, O, and C elements. In the case of CoO/CoP, in addition to the aforementioned elements, an anticipated P element is also evident. The absence of other elemental peaks attests to the high purity of the samples. The high-resolution XPS spectra of the Co 2p orbital for Co_3_O_4_ and CoO/CoP are illustrated in Fig. 3a and b, respectively. In the case of Co_3_O_4_, two sets of doublets are deconvoluted. The peaks at 780.0 and 795.2 eV pertain to the Co^3+^ 2p_3/2_ and 2p_1/2_ orbitals, respectively.^66,67^ A second set of peaks at 781.2 and 796.6 eV corresponds to the Co^2+^ 2p_3/2_ and 2p_1/2_ orbitals, respectively. The intensity ratio of Co^3+^ : Co^2+^ is determined to be 1.92 : 1, closely mirroring the nominal ratio for Co_3_O_4_. Conversely, in the case of CoO/CoP, the Co^3+^ peaks are absent, implying a thorough transformation of Co_3_O_4_ into other phases following phosphorization. The intensity of the Co^3+^ peaks increases significantly, indicating that a portion of Co_3_O_4_ has transformed into CoO. This inference is further corroborated by the examination of satellite peaks, where the satellite peaks in CoO are more observable compared to those in Co_3_O_4_. This observation aligns with the known phenomenon that the 2+ oxidation state of Co exhibits more prominent satellite peaks than other oxidation states.^68^ Furthermore, a new set of peaks at 778.5 and 793.4 eV emerges, aligning with the Co^0^ 2p_3/2_ and 2p_1/2_ orbitals, respectively. The presence of Co^0^ unequivocally signifies the transformation of some portion of Co_3_O_4_ into CoP. The XPS analysis thus provides further confirmation of the existence of the CoO/CoP heterointerface. Meanwhile, a chemical shift of +0.6 eV is observed for the Co^2+^ 2p_3/2_ orbital after phosphorization, which signifies a downshift of the valence state of Co, indicating electron transfer from CoP to CoO across the heterointerface. The high-resolution O 1s XPS spectra of Co_3_O_4_ and CoO/CoP are depicted in Fig. 3c and d, respectively. The O 1s spectrum is resolved into two distinct peaks. The peak at 530 eV is indicative of O–metal bonds, while the peak at 531.2 eV corresponds to O–H bonds arising from adsorbed surface hydroxyls.^69,70^Fig. 3e and f present the high-resolution P 2p XPS spectra. As expected, P signal is absent in Co_3_O_4_. On the contrary, two prominent peaks can be identified for CoO/CoP. The peak at 130 eV is attributed to the P–Co bond in CoP, while the peak at 134 eV is ascribed to the P–O bond.^71,72^

**Fig. 4:**
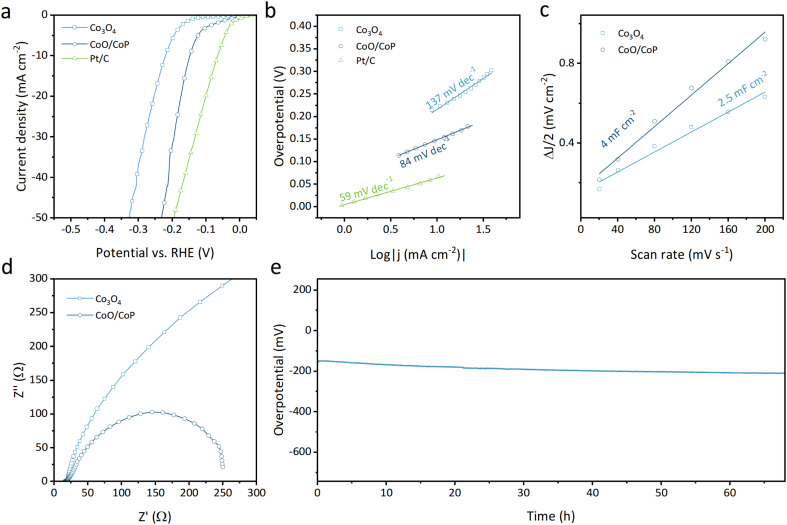
(a) LSV curves of Co_3_O_4_ and CoO/CoP, respectively. (b) Tafel slopes of Co_3_O_4_ and CoO/CoP, respectively. (c) *C*_dl_ of Co_3_O_4_ and CoO/CoP, respectively. (d) EIS of Co_3_O_4_ and CoO/CoP, respectively. (e) Stability test.

The electrocatalytic activity for the hydrogen evolution reaction (HER) of the synthesized samples was assessed using an electrochemical workstation. As illustrated in Fig. 4a, the linear sweep voltammetry (LSV) curves depict the Faraday current as a function of overpotential for Co_3_O_4_, CoO/CoP, and Pt/C (wt. 20%) powders. Notably, Co_3_O_4_ requires an overpotential of 220 mV to achieve a current density of 10 mA cm^−2^, whereas CoO/CoP demonstrates a significantly reduced overpotential of 148 mV. This substantial reduction indicates that the formation of the CoO/CoP heterointerface greatly enhances the HER activity. On one hand, the formation of CoO/CoP can facilitate efficient electron transfer between CoO and CoP, which is essential for the electrochemical reduction of protons to hydrogen. This improved electron transfer can accelerate the kinetics of HER and lower the overpotential. On the other hand, the CoO/CoP heterointerface can lead to changes in the local coordination environment of Co ions, which can influence their intrinsic catalytic activity by altering their electronic structure. The Tafel slopes for the respective materials, derived from the LSV curves, are presented in Fig. 4b. The Tafel slope for Co_3_O_4_ is measured at 137 mV dec^−1^, whereas that for CoO/CoP is 84 mV dec^−1^. These results suggest that the formation of the CoO/CoP heterointerface considerably reduces the kinetic barrier in the HER process. To delve into the underlying physics of the enhanced HER activity of CoO/CoP, the double layer capacity (*C*_dl_) was measured using cyclic voltage methods within the non-Faraday range, as presented in Fig. 4c and S5.† Specifically, CoO/CoP exhibits a *C*_dl_ of 4 mF cm^−2^, surpassing that of Co_3_O_4_ (2.5 mF cm^−2^). This indicates that CoO/CoP possesses a higher electrochemically active area than Co_3_O_4_ after phosphorization. Electrochemical impedance spectra were also recorded and are depicted in Fig. 4d. CoO/CoP exhibits significantly lower impedance compared to Co_3_O_4_, signifying faster charge transfer at the reaction surface. The measured electrochemical properties are summarized in Table S1.† The stability of a catalyst is also a crucial parameter for evaluating its performance. The long-term stability test of CoO/CoP was conducted at a current density of 10 mA cm^−2^ for over 65 hours (Fig. 4e).

**Fig. 5:**
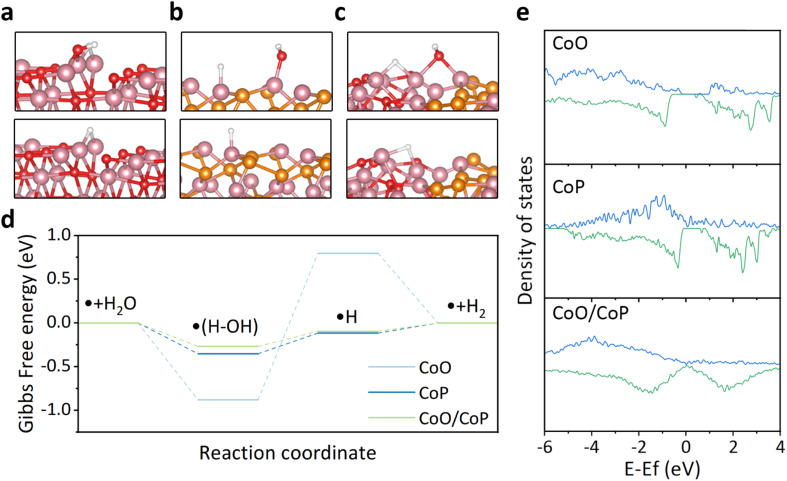
(a–c) DFT calculation structure model for CoO, CoP and CoO/CoP, respectively. (d) Corresponding Gibbs free energy change during HER in alkali. (e) Partial density of states of Co in CoO, CoP and CoO/CoP, respectively. The bule lines indicate spin-up states while the green lines indicate spin-down states.

To explore the underlying reasons behind the improved hydrogen evolution reaction (HER) activity of CoO/CoP, theoretical calculations based on the density functional theory (DFT) were conducted. The CoO/CoP heterointerface was constructed and relaxed, in comparison with individual CoO and CoP, as depicted in Fig. S5 and S6.† In alkaline environments, the HER process involves two main steps: the initial dissociation of water and the subsequent desorption of hydrogen from the catalyst surface. The optimized structures for water dissociation and hydrogen desorption for the three considered models are shown in Fig. 5a–c, respectively. The reaction barrier, represented by the Gibbs free energy change, for these models is illustrated in Fig. 5d. It is observed that CoO exhibits excessively strong adsorption for water, leading to a substantial barrier for water dissociation. In contrast, the CoO/CoP heterostructure exhibits a minimal reaction barrier for both water dissociation and hydrogen desorption. The energy barrier of CoP falls between the barriers observed for CoO and CoO/CoP. To elucidate the optimized reaction barrier of the CoO/CoP heterostructure, the partial density of states of the active Co site in the three models was calculated, as illustrated in Fig. 5e. The analysis reveals a large band gap in CoO. The band gap disappears for the up-spin states in CoP, while it persists in the down-spin band. In the case of the CoO/CoP heterostructure, the band gap disappears for both up-spin and down-spin bands. Therefore, the interaction between CoO and CoP leads to the elimination of the bandgap, enhancing the interaction between the catalyst and reaction radicals.

## 4. Conclusion

In conclusion, a facile phosphorization process has been successfully employed to achieve the *in situ* transformation from a single-phase Co_3_O_4_ to CoO/CoP hybrid phases. The synthesized samples have undergone comprehensive characterization using various techniques, including XRD, BET, SEM, TEM, among others. Throughout the phase transformation, the mesoporous nature of Co_3_O_4_ has been retained, resulting in the formation of porous CoO/CoP heterointerfaces. The presence of strong charge transfer across the CoO/CoP heterointerface suggests a robust interaction between the hybrid phases. Importantly, CoO/CoP demonstrates significantly enhanced catalytic activity for HER compared to pristine Co_3_O_4_. DFT calculations offer insights into the observed high HER activity, revealing that the elimination of the band gap in the spin-down band of Co in CoO/CoP contributes to the improved catalytic performance.

## Conflicts of interest

The authors declare no conflict of interests regarding the publication of this article.

## Supplementary Material

RA-014-D4RA02063D-s001
