# Supplementary material for: An in situ phase transformation induced mesoporous heterointerface for the alkaline hydrogen evolution reaction
Source: RSC Adv. 2024 May 7;14(21):14886–93. doi: 10.1039/d4ra02063d (PMC11075778; doi:10.1039/d4ra02063d)
Supplement: RA-014-D4RA02063D-s001 [file RA-014-D4RA02063D-s001.pdf]

Electronic Supplementary Material (ESI) for RSC Advances.
This journal is © The Royal Society of Chemistry 2024

Supporting Information

DFT calculations method

We utilized the Vienna Ab Initio Package (VASP) [1,2] to conduct all spin-polarized density functional theory (DFT) calculations within the generalized gradient approximation (GGA), employing the Perdew-Burke-Ernzerhof (PBE) [3] formulation. The projected augmented wave (PAW) potentials [4,5] were selected to model the ionic cores, and valence electrons were treated within a plane wave basis set with a kinetic energy cutoff of 450 eV. The electronic energy was considered self-consistent when the energy change fell below 10^{-5} eV. For geometry optimization, convergence was achieved when the energy change was less than 0.04 eV $Å^{-1}$. The vacuum spacing in a direction perpendicular to the structure's plane was set at 18 Å. The weak interaction was accounted for by applying the DFT+D3 method, employing an empirical correction based on Grimme's scheme [6, 7]. The Gibbs free energy for each elementary step was calculated as $G = E_{elec} + E_{ZPE} - TS$, where E_{elec} is the electronic energy at 0 K calculated by DFT, E_{ZPE} is the zero-point energy term, and T is the absolute temperature (here 298.15 K), S is the entropy.

References

1. Kresse, G.; Furthmüller, J. Comput. Mater. Sci. 1996, 6, 15−50.
2. Kresse, G.; Furthmüller, J. Phys. Rev. B 1996, 54, 11169−11186.
3. Perdew, J. P.; Burke, K.; Ernzerhof, M. Generalized Gradient Approximation Made Simple. Phys. Rev. Lett. 1996, 77, 3865−3868.
4. Kresse, G.; Joubert, D. From Ultrasoft Pseudopotentials to the Projector Augmented-Wave Method. Phys. Rev. B 1999, 59, 1758-1775.
5. Blöchl, P. E. Projector Augmented-Wave Method. Phys. Rev. B 1994, 50, 17953-17979.
6. S. Grimme, J. Antony, S. Ehrlich, and S. Krieg, J. Chem. Phys. 2010， 132, 154104.
7. S. Grimme, S. Ehrlich, and L. Goerigk, J. Comp. Chem. 2011， 32, 1456.

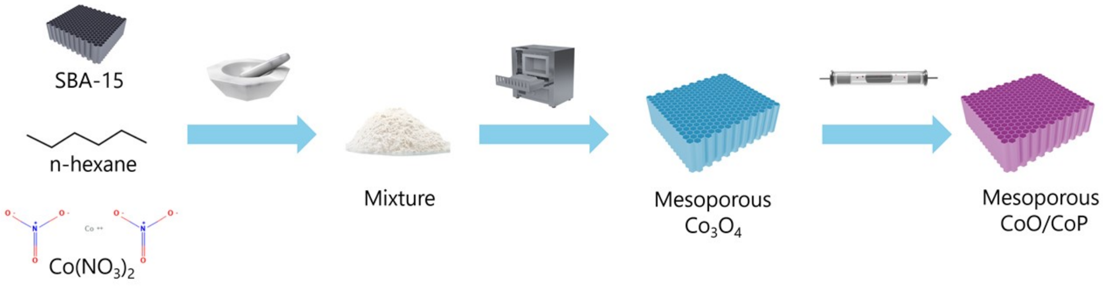

Figure S1. Synthesis scheme of mesoporous CoO/CoP.

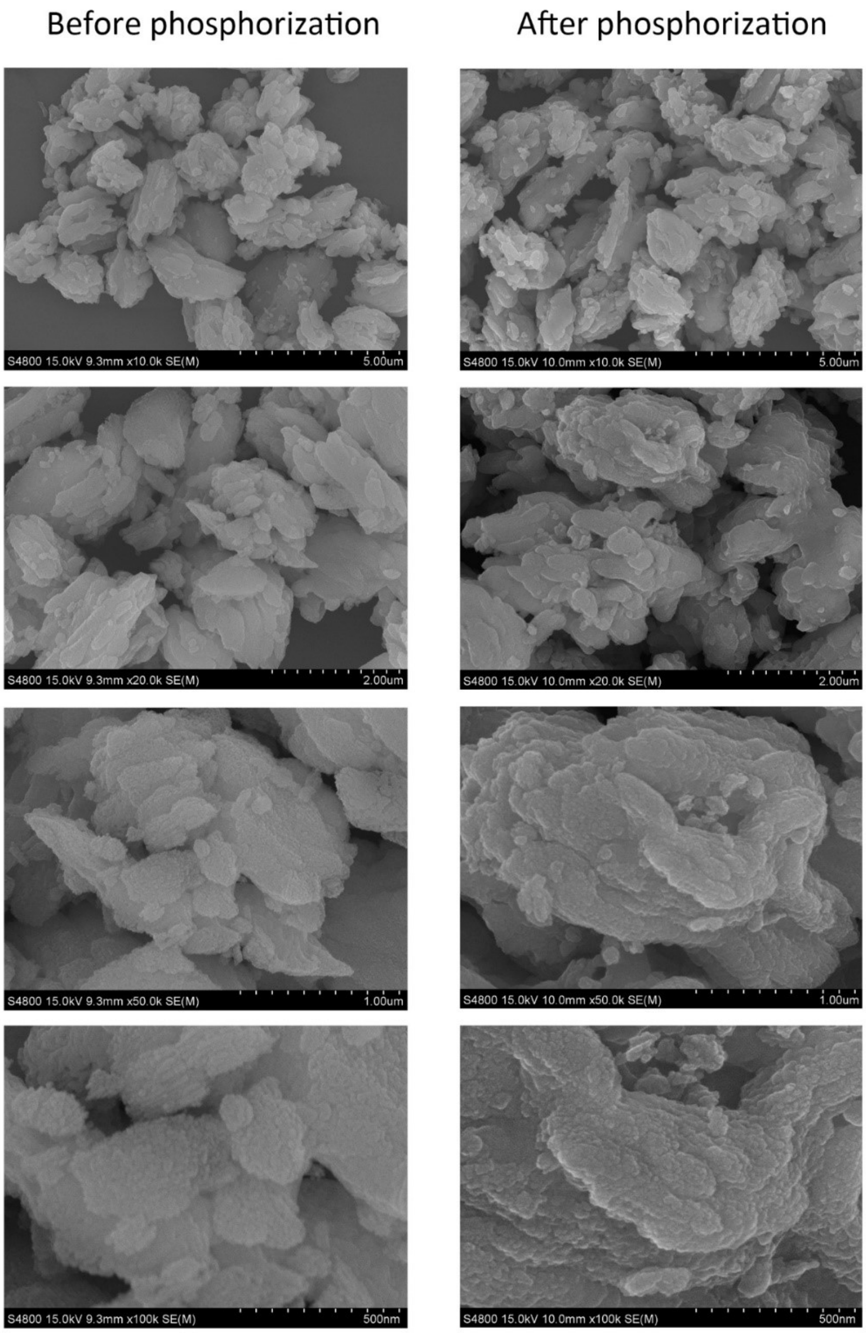

Figure S2. SEM photos of Co_3O_4 and Co3O4-P at various magnifications.

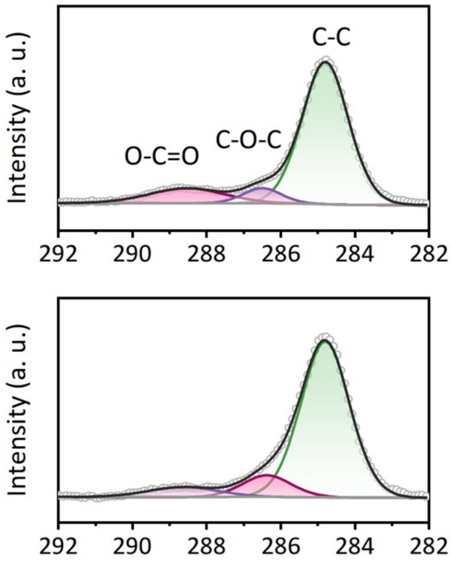

Figure S3. C 1s XPS spectra of Co_3O_4 and Co_3O_4-P, respectively.

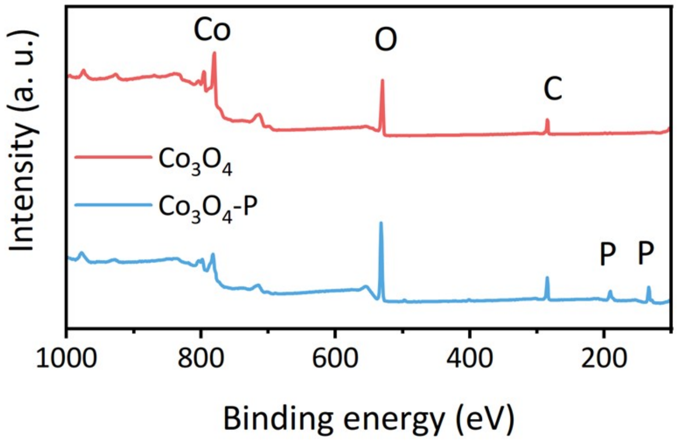

Figure S4. XPS survey of Co_3O_4 and Co_3O_4-P, respectively.

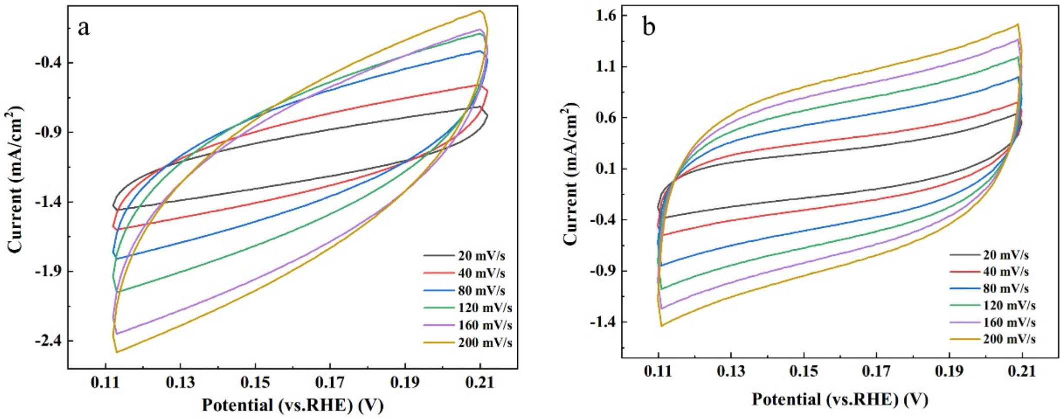

Figure S5. CV sweeps of Co_3O_4 and Co_3O_4-P, respectively.

Table S1. Summary of the electrochemical data of various electrocatalysts in 1 M KOH.

Sample	HER Activity		C_{dl} (mF cm^{-2})	EIS	
	η_{10} (mV)	Tafel Slope (mV dec^{-1})		R_s (Ω)	R_{ct} (Ω)
Co_3O_4	220	137	4	17.7	830.9
Co_3O_4-P	148	84	2.5	17.9	276.5

η_{10}: the overpotential at the current density of 10 mA cm^{-2}; C_{dl}: double layer capacitance; R_s and R_{ct}: electrolyte resistivity and electron transfer resistivity fitted from EIS analysis, respectively.

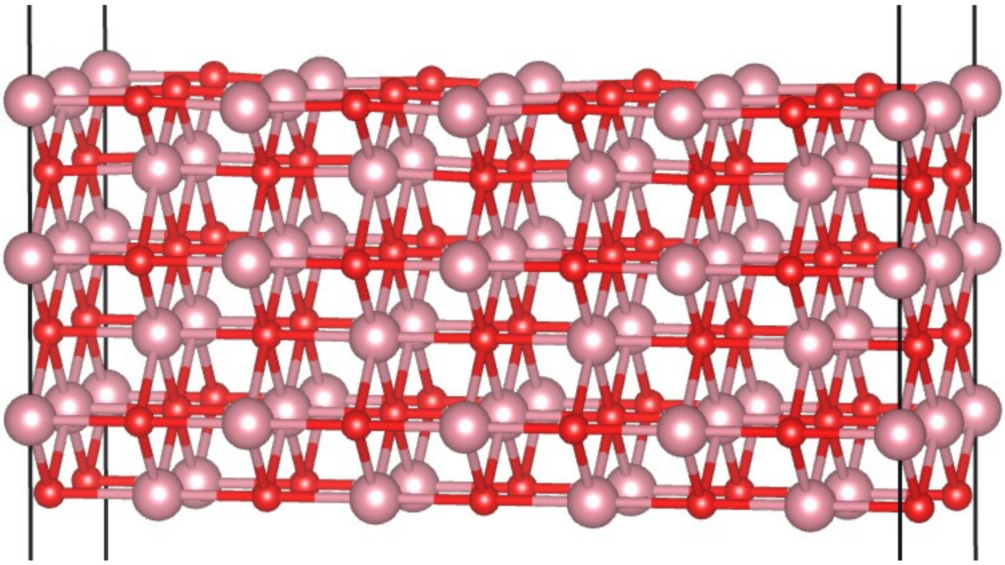

Figure S6. DFT calculation model for CoO.

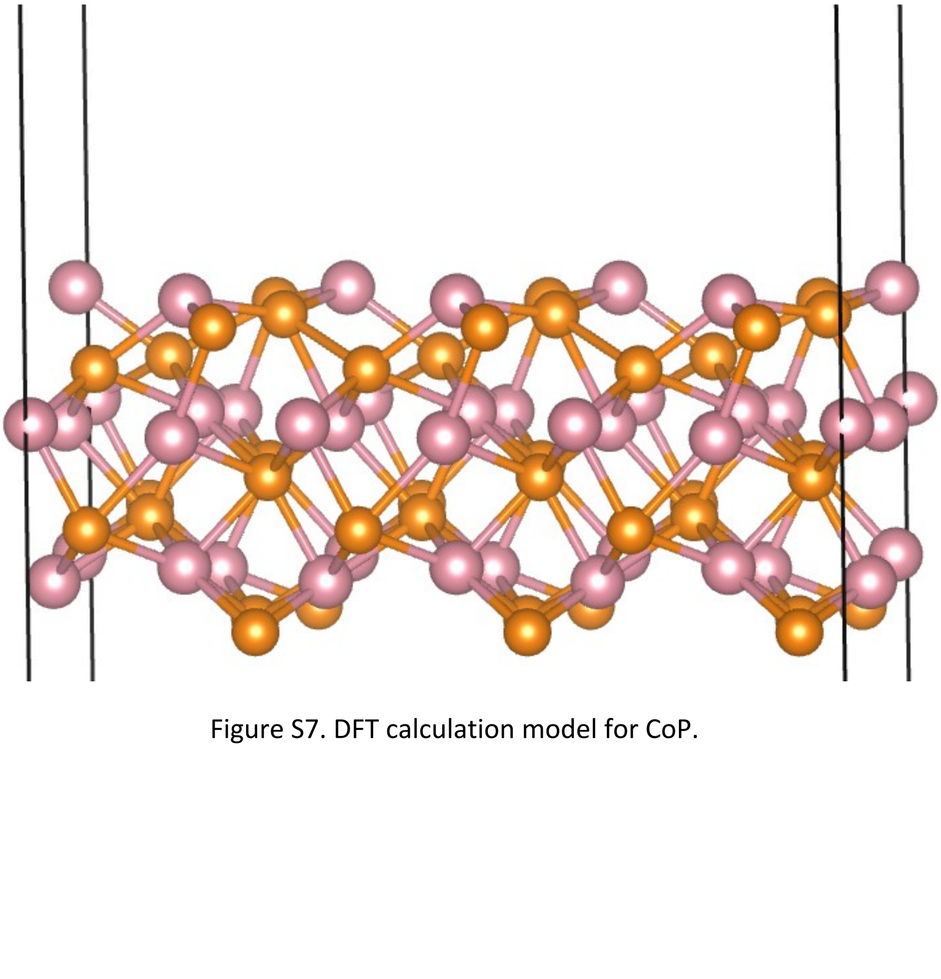

Figure S7. DFT calculation model for CoP.

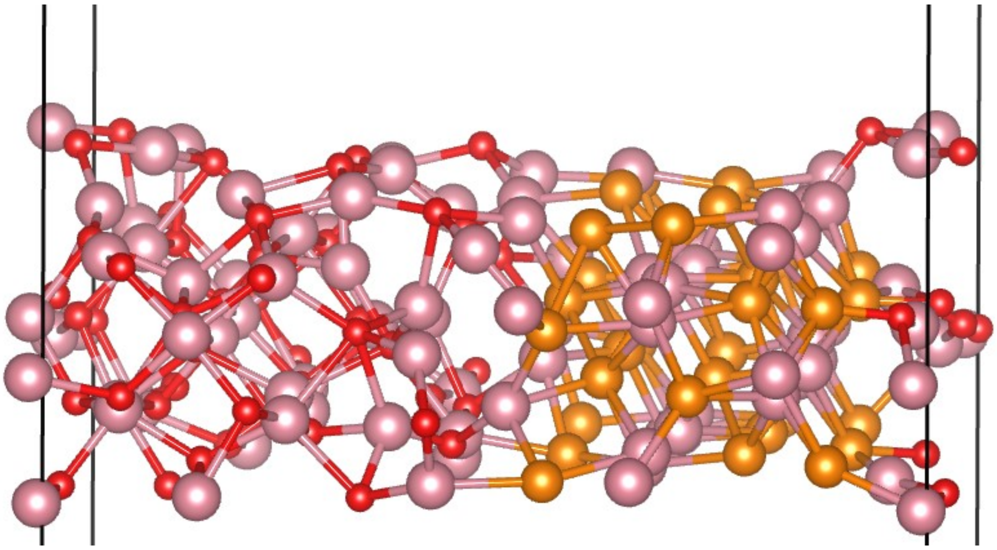

Figure S8. DFT calculation model for Co_3O_4-P.
